# What Works for One May Not Work for Another: A New Warning for Modafinil

**DOI:** 10.7759/cureus.27287

**Published:** 2022-07-26

**Authors:** Harim Kim, Girma M Ayele, Rediet T Atalay, Siham Hussien, Bereket Tewoldemedhin, Miriam B Michael, Steven M Scharf

**Affiliations:** 1 Internal Medicine, University of Maryland School of Medicine, Baltimore, USA; 2 Internal Medicine, Howard University Hospital, Washington DC, USA; 3 Internal Medicine, Lower Bucks Hospital, Bristol, USA; 4 Internal Medicine, Howard University Hospital, Washingon DC, USA; 5 Medicine, University of Maryland School of Medicine, Baltimore, USA

**Keywords:** sodium oxybate for narcolepsy, modafinil and palpitation, modafinil side effect, modafinil, narcolepsy treatment, narcolepsy

## Abstract

Narcolepsy is a clinical syndrome of hypothalamic disorder characterized by several sleep-wake disorders. The most common features include daytime sleepiness associated with hallucinations (hypnagogic and hypnopompic hallucinations) at the transition time of sleep-wake time, cataplexy or sudden loss of muscle tone, and sleep paralysis. We present a case of a patient affected with both narcolepsy and postural orthostatic tachycardia syndrome (POTS). POTS is a rare disorder characterized by orthostatic intolerance and abnormal autonomic response while sustaining an upright posture. In this case report, we highlight the impact of POTS on the choice of pharmacotherapy for narcolepsy.

## Introduction

Narcolepsy is a debilitating neurological disorder presumed to originate in the hypothalamic region of the brain and requires lifelong treatment [1]. The core symptoms include excessive daytime sleepiness (EDS), vivid dreams and hallucinations at the transition time of sleep and waking up, also known as hypnagogic/hypnopompic hallucinations, sleep paralysis, and frequently disrupted night-time sleep. Two subcategories include EDS with cataplexy or low hypocretin level, and the second type is EDS without cataplexy with normal hypocretin levels [2]. Psychostimulants are the first-line treatment recommended for narcolepsy, having side effects including palpitation, which our patient experienced [3,4]. Our patient had an increased chance of this side effect due to her underlying medical condition known as postural orthostatic tachycardia syndrome (POTS). POTS is an autonomic dysfunction that presents with palpitation and tremors [5]. These symptoms were exacerbated by using psychostimulants for narcolepsy. Changing the medication to sodium oxybate improved the patient's narcolepsy symptoms and alleviated the palpitation.

## Case presentation

The patient is a 47-year-old female with a history of POTS diagnosed approximately 13 years ago when she presented with syncope and exercise intolerance symptoms. At that time, hypotension due to hypovolemia, adrenal insufficiency, as well as secondary causes of autonomic neuropathy were ruled out. Physical examination revealed a pulse of 73 beats per minute (bpm) and elevated blood pressure of 127/79 mmHg with no orthostatic changes. Lab testing was within the normal range. She was referred to the sleep clinic for evaluation of EDS that she had been experiencing for several years, associated with multiple awakenings during the night and recent episodes of a sudden loss of muscle tone in the neck. The diagnosis of narcolepsy was confirmed with overnight polysomnography and multiple sleep latency tests. 

**Figure 1:**
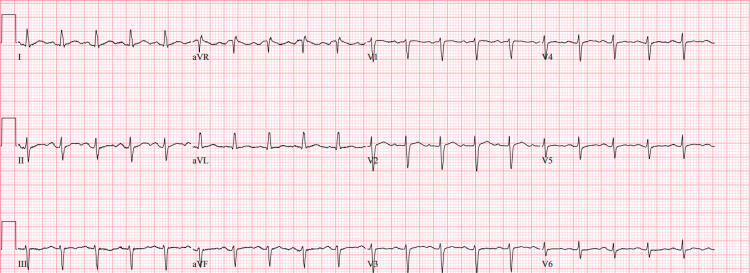
EKG of the patient while on modafinil showing sinus tachycardia with a ventricular rate of 121 beats per minute.

She was started on modafinil 200 mg daily. A month into the treatment, the patient reportedly experienced nausea and headaches. She visited the ED after experiencing several episodes of palpitations and chest pain. Repeated EKGs were done and showed sinus tachycardia (Figure 1). Other laboratories like d-dimer, thyroid hormone, complete blood count, electrolytes, and troponin were all unremarkable. It was decided to switch the medication to sodium oxybate, and the patient was then placed on Holter monitoring. The patient reported resolution of her symptoms, and the Holter monitor did not record any abnormal cardiac events. After switching from modafinil to sodium oxybate, the patient's sleep quality improved significantly, and episodes of tachycardia and palpitations resolved.

## Discussion

Narcolepsy is one of the most common causes of daytime sleepiness. The cause of narcolepsy has been under investigation for more than 150 years, but more groundbreaking discoveries have been made, and a clear cause has been identified in the past 20 years. Two studies that were done in 1998 have independently identified neuropeptides (orexin A and orexin B) that are only produced in the lateral hypothalamus of the brain to be associated with narcolepsy. These neuropeptides function as excitatory hormones by binding to their respective receptors. Subsequent research has found low orexin levels in the cerebrospinal fluid and neurons in individuals with narcolepsy [6]. The orexin neurons innervate several regions of the brain that promote wakefulness and regions that suppress sleep, particularly rapid eye movement (REM) sleep. In addition, many studies show that orexin increases the level of gamma-aminobutyric acid (GABA) and glutamate by increasing the level of cytoplasmic calcium, which synergizes with the excitatory effects of orexin [7]. 

The neurons that produce orexin are only found in the lateral hypothalamus and have several projections to brain regions that are responsible for arousal and decreasing REM sleep, like locus coeruleus (LC), dorsal raphe (DR), periaqueductal grey (PAG), tuberomammillary nucleus (TMN), basal forebrain, and spinal cord. In addition, they also innervate regions that are responsible for feeding, metabolism, and autonomic tone [7].

Two types of narcolepsy have been identified: narcolepsy type 1 and narcolepsy type 2, also called narcolepsy with and without cataplexy, respectively. Type 1 narcolepsy occurs due to the complete loss of all neurons called hypocretin, which contains orexin. Even though the cause is not straightforward, the autoimmune process may play a critical role [8]. Approximately 95% of patients with type 1 narcolepsy have HLA haplotype DQB1*0602. The cause of narcolepsy type 2 is not known clearly, but recent studies suggest that impaired receptors and incomplete destruction of orexin cells may be the cause of type 2 narcolepsy [8]. 

To diagnose narcolepsy, patients should have at least 6 hours of sleep for at least two weeks, which is confirmed by using actigraphy with a sleep log. If patients get at least 6 hours of sleep, polysomnography (PSG) is done to rule out other sleep disorders. The next step is to do the multiple sleep latency test (MSLT), in which patients are given multiple 20-minute naps lasting 2 hours. A test is positive if the onset of the REM sleep is less than 15 minutes at least twice and the short mean sleep latency is less than 8 minutes. Finally, type 1 narcolepsy can be diagnosed with a low cerebrospinal fluid (CSF) level of hypocretin-1 (<110 pg/ml) [8].

Narcolepsy treatment aims at achieving a state of alertness during conventional wake hours to increase the functionality of the individual [2]. Depending on the medication class used, the effects can be immediate as in the case of modafinil and amphetamines, or it may take several days before the desired response is seen, as typical with oxybates [2]. Medication selection for these patients is highly individualized and is based on symptom severity and predominating symptoms (somnolence vs. cataplexy). This, in turn, dictates medication efficacy, patient’s age, comorbidities, side effect profiles, and cost of each medication [5]. Other treatment modalities include psychosocial support, including coping with disease misconceptions and addressing safety and medication risks. Patients are also regularly counseled on scheduled naps and good sleep hygiene [9].

When it comes to medication selection, most drugs target either daytime sleepiness or cataplexy [9]. Ideally, a single drug regimen is preferred. However, if symptom control is inadequate or side effects arise, a second agent is added, and the first medication is titrated and removed [2,9]. Modafinil is one of the first-line pharmacologic therapy for severe daytime sleepiness, a stimulant that works by blocking dopamine reuptake [3]. Common adverse effects include headaches, nervousness, anxiety, nausea, anorexia, palpitations, and insomnia [2,4]. 

Sodium oxybate is a second-line therapy for cataplexy, a metabolite of GABA [10]. It is a slow-acting drug with beneficial effects for narcolepsy occurring gradually over a one-month period [8,9]. It is highly known for its sedating and retrograde amnestic properties and potential for misuse [3,7]. It has also been used for sexual assault as a “date rape” drug [9]. Other side effects noted include respiratory depression and contraindicated in existing sleep apnea patients [2].

Other stimulants are used, such as solriamfetol, a selective dopamine and norepinephrine reuptake inhibitor with wake-promoting effects [3]. They are also used but are contraindicated in patients that are taking or were recently (<14 days) taking monoamine oxidase inhibitors due to the risk of hypertensive reaction [1,4,10]. Drugs like venlafaxine, fluoxetine, and atomoxetine are first-line therapy for cataplexy [10]. REM suppressing agents such as selective serotonin reuptake inhibitors (SSRIs) and tricyclic antidepressants (TCAs) may reduce cataplexy. However, their use for this condition remains off-label and is currently not approved by the FDA [5,9]. Abrupt withdrawal of these agents can lead to rebound severe cataplexy and, in severe cases, status cataplecticus [2].

The other medical problem our patient had was POTS. POTS was identified in 1921 and was thought to be only due to autonomic dysfunction. However, recent studies suggest cardiac deconditioning, increased sensitivity to beta-adrenoreceptors, and neuropathy in the distal vessels are also responsible for POTS. Symptoms are vital clues to diagnosing POTS. Symptoms include postural symptoms like palpitation, tremor, weakness, blurred vision, and exercise intolerance or nonpostural symptoms like abdominal pain, bloating, nausea, fatigue, sleep issues, and migraine [6].

One study that was done by Bosco A et al. evaluated the effect of psychostimulants used for the treatment of narcolepsy type I on blood pressure (BP) and heart rate (HR). The study concluded that patients treated with psychostimulants compared to control patients had twice higher HR and BP [5]. Based on this result, we can imagine the effect of psychostimulants on HR in patients with POTS. Therefore, we strongly suggest that when choosing treatment for patients with narcolepsy, patients’ HR should be put under consideration. 

Few case reports are associated with modafinil and cardiac arrhythmia. One case reported 11 attacks of non-sustained ventricular tachycardia in young patients who abused modafinil with no structural heart disease [11]. Another case reported polymorphic ventricular tachycardia in a 50-year-old patient with narcolepsy and no structural heart disease after he was started on modafinil [12]. In both cases, patients were investigated, and all possible causes of arrhythmia were ruled out, and the arrhythmia subsided after discontinuation of modafinil [11,12]. 

We have reviewed the United States FDA Adverse Event Reporting System (FAERS) database. The search revealed 11 cases of arrhythmia and one death due to fetal arrhythmia. However, these data are individual-based, and a causal association between cause and effect cannot be established [13].

## Conclusions

This case illustrates a new potential warning for a class of medications for narcolepsy treatment. Central-acting stimulants like modafinil and solriamfetol are usually considered the first line of treatment. However, like in this case, the stimulant effect can augment dysautonomia and cause side effects like tachycardia/arrhythmia in a patient with cardiovascular risk factors. Sodium oxybate is a metabolite of GABA and is an alternative medication for patients who cannot tolerate stimulants. Therefore, we suggest that the safety and use of stimulants as a first-line treatment should be strongly under reconsideration for patients with cardiac conditions like POTS and arrhythmia.
