# Correction: What Works for One May Not Work for Another: A New Warning for Modafinil

**DOI:** 10.7759/cureus.c69

**Published:** 2022-08-08

**Authors:** Harim Kim, Girma M Ayele, Rediet T Atalay, Siham Hussien, Bereket Tewoldemedhin, Miriam B Michael, Steven M Scharf

**Affiliations:** 1 Internal Medicine, University of Maryland School of Medicine, Baltimore, USA; 2 Internal Medicine, Howard University Hospital, Washington DC, USA; 3 Internal Medicine, Lower Bucks Hospital, Bristol, USA; 4 Internal Medicine, Howard University Hospital, Washingon DC, USA; 5 Medicine, University of Maryland School of Medicine, Baltimore, USA

This article has been corrected to include Steven M. Scharf as last author. Dr. Scharf was accidentally omitted from the author list by the submitting author and this mistake was not noticed until after publication. The authors regret this error.

